# Unusual Presentation of Acute Appendicitis: A Case Report of de Garengeot Hernia

**DOI:** 10.7759/cureus.90397

**Published:** 2025-08-18

**Authors:** Hovhannes Hayrapetyan, Yixiang Zhan, Dharshan Vummidi

**Affiliations:** 1 Department of Radiology, Wayne State University School of Medicine, Detroit, USA; 2 Department of Radiology, Henry Ford Health System, Detroit, USA

**Keywords:** appendicitis in femoral hernia, canal of nuck hydrocele, ct abdomen and pelvis, de garengeot hernia, groin hernia imaging, preoperative radiological diagnosis, radiological misdiagnosis, radiologic diagnosis hernia, strangulated femoral hernia, ultrasound abdomen and pelvis

## Abstract

A de Garengeot hernia (DGH) is a rare anatomic variant involving the herniation of the appendix through the femoral canal. It is an uncommon surgical entity with diagnostic challenges. We report the case of a 63-year-old woman who presented with right inguinal region pain and swelling, in which preoperative ultrasound (US) and computed tomography (CT) identified the herniated, inflamed appendix within the canal of Nuck, which was intraoperatively found to be rather a femoral hernia. This rare case illustrates the characteristic imaging features of DGH and highlights the importance of early and accurate radiologic diagnosis for surgical planning and improved patient outcomes.

## Introduction

A de Garengeot hernia (DGH) is a rare variant of femoral hernia, which involves the herniation of the appendix within the femoral canal and was first described in 1731 [1]. This entity comprises approximately 0.5-5% of all femoral hernia cases and is often preoperatively misdiagnosed as an incarcerated or a strangulated femoral hernia and less commonly as an inguinal hernia due to nonspecific presentation [2]. The presence of an inflamed appendix within the hernia sac is especially uncommon and found in approximately 0.08-0.13% of cases [3]. According to a systematic review by Guenther et al., early identification of DGH through imaging was achieved in only 31.5% of cases [4]. The risks of appendiceal inflammation and potential complications highlight the importance of establishing the radiological diagnosis early on. This case illustrates valuable clinical insight into identifying this type of rare hernia, particularly by demonstrating its appearance on ultrasound (US) and computed tomography (CT), for accurate diagnosis and supporting optimal surgical intervention.

## Case presentation

**Figure 1:**
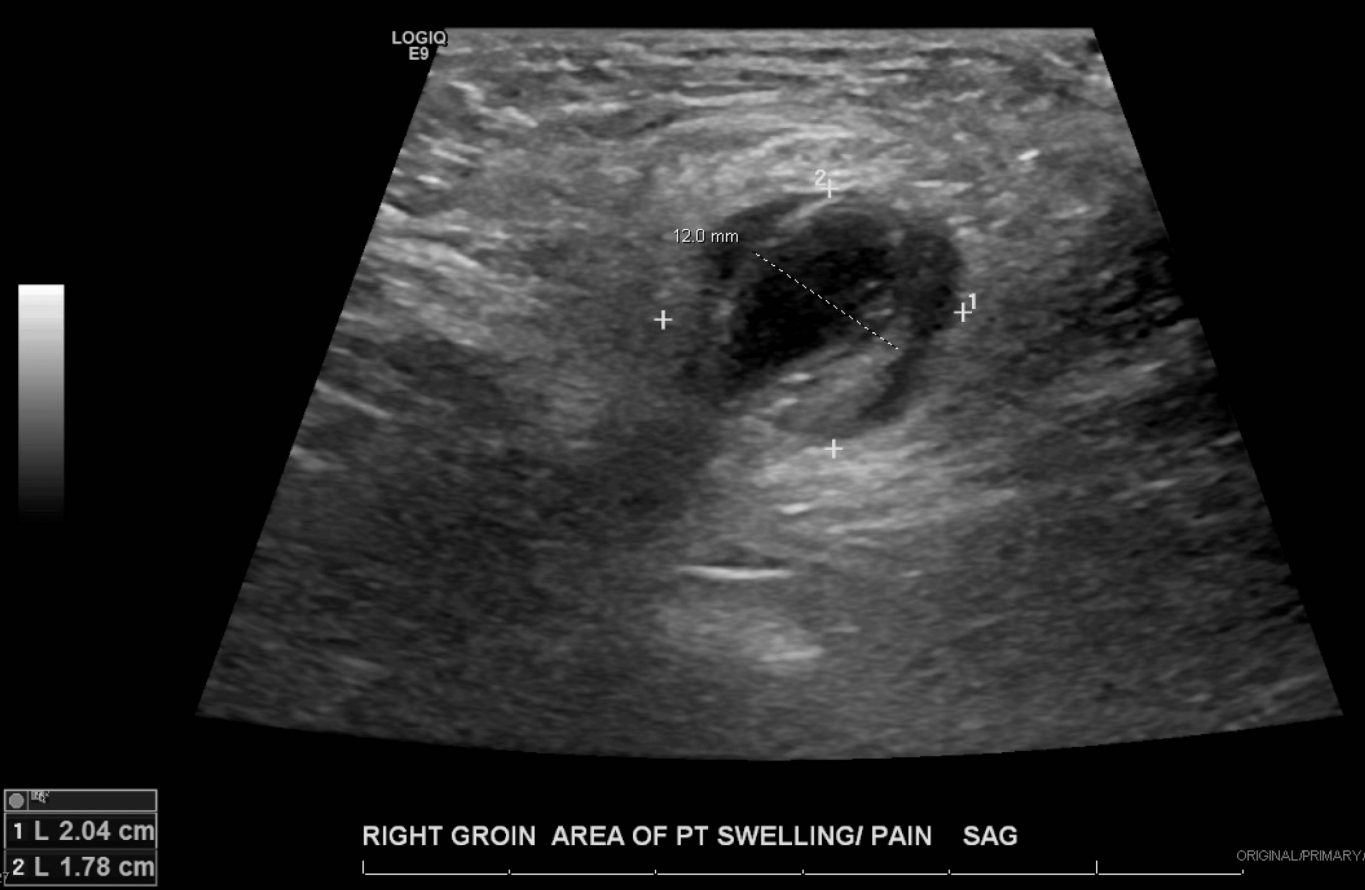
Ultrasound of the right groin Sagittal view of the hernia, which measures 2.04×1.78 cm, containing the appendix, which measures 12 mm.

**Figure 2:**
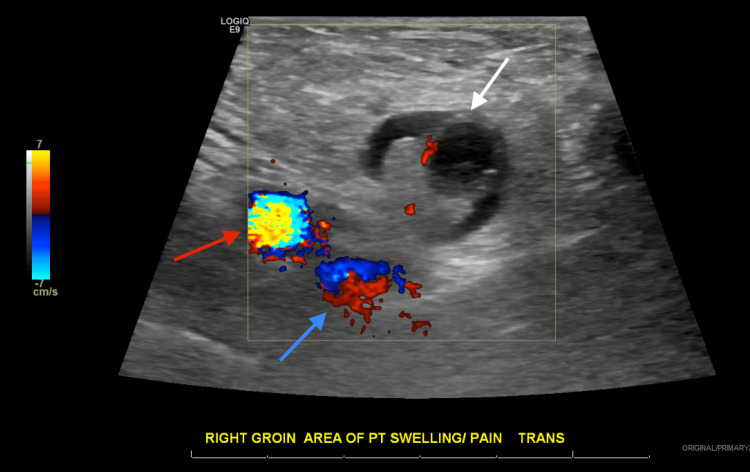
Ultrasound of the right groin Transverse view with color Doppler showing the femoral artery (red solid arrow) and vein (blue solid arrow) in relation to the hernia (white solid arrow).

**Figure 3:**
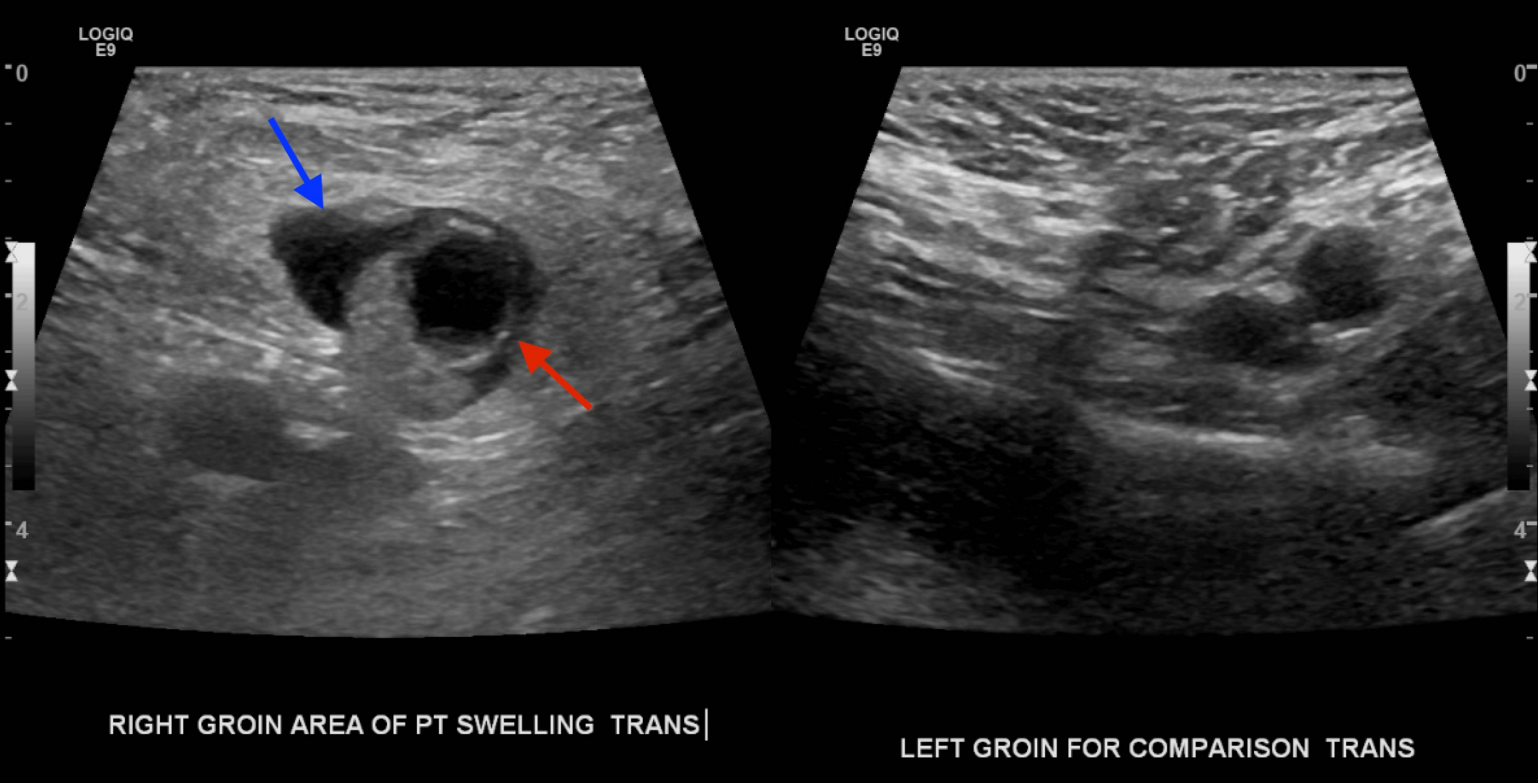
Ultrasound of the right and left groin Comparison of the transverse view of the right and left groin regions with the femoral hernia (blue solid arrow) and a cystic structure within (red solid arrow) present in the right groin.

**Figure 4:**
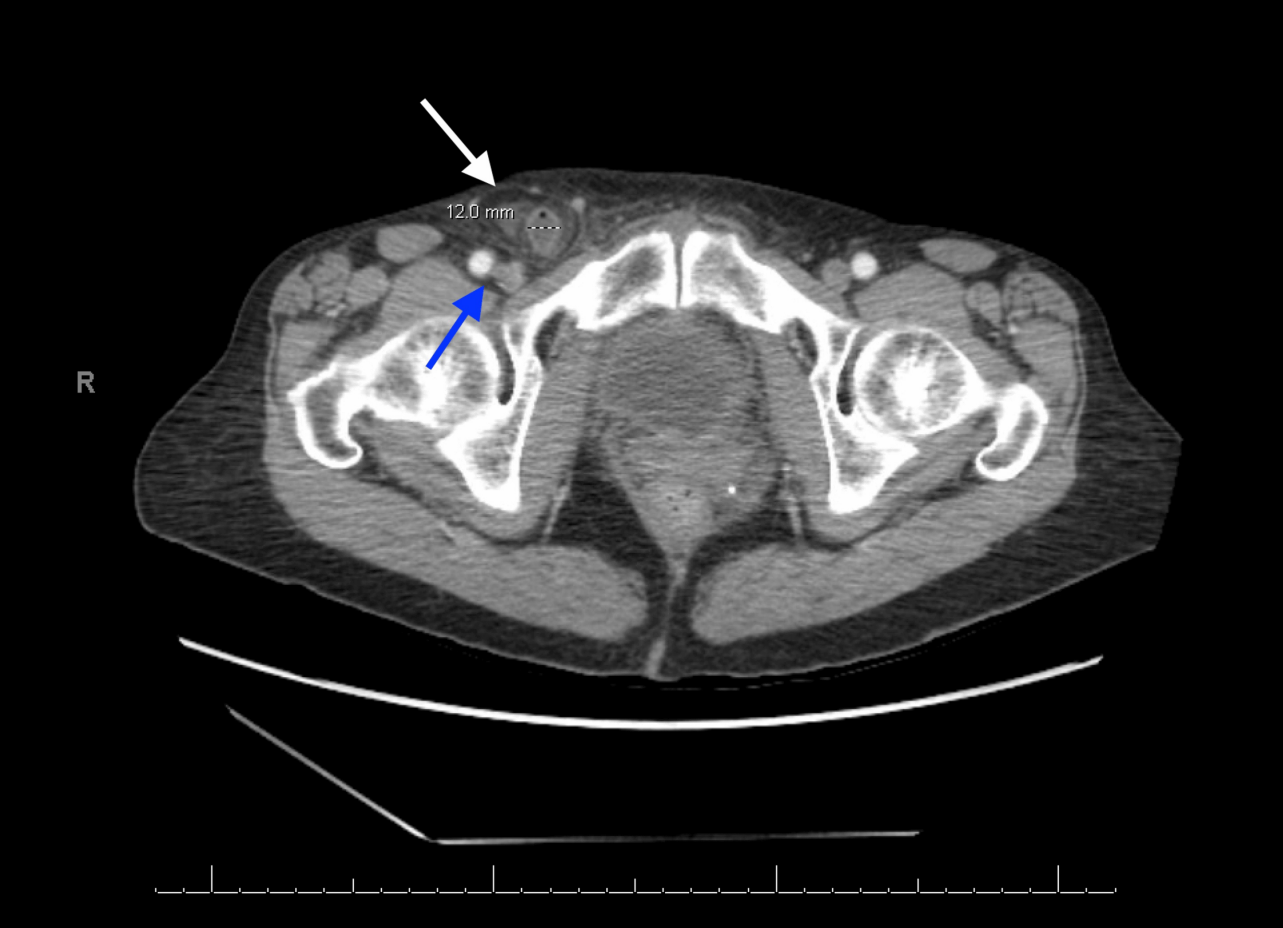
Contrast-enhanced computed tomography of the abdomen and pelvis Axial view illustrating the right femoral hernia (white solid arrow), containing the appendix which measures 12 mm, medial to the femoral blood vessels (blue solid arrow).

**Figure 5:**
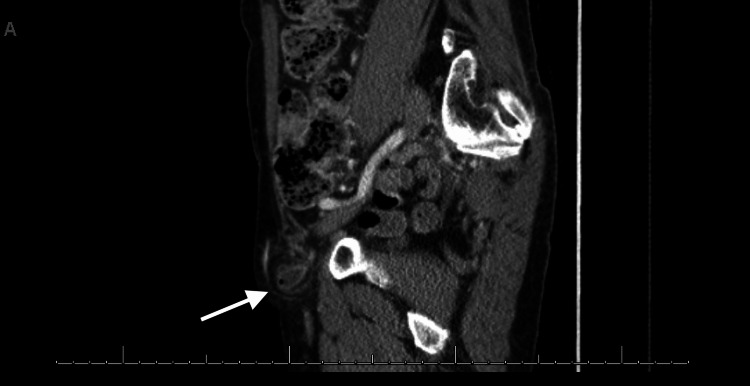
Contrast-enhanced computed tomography of the abdomen and pelvis Sagittal view showing the right femoral hernia with abrupt caliber change of the distal appendix as it enters the hernia sac (white solid arrow). Small amount of reactive free fluid within the hernia sac with associated acute appendicitis secondary to proximal obstruction.

**Figure 6:**
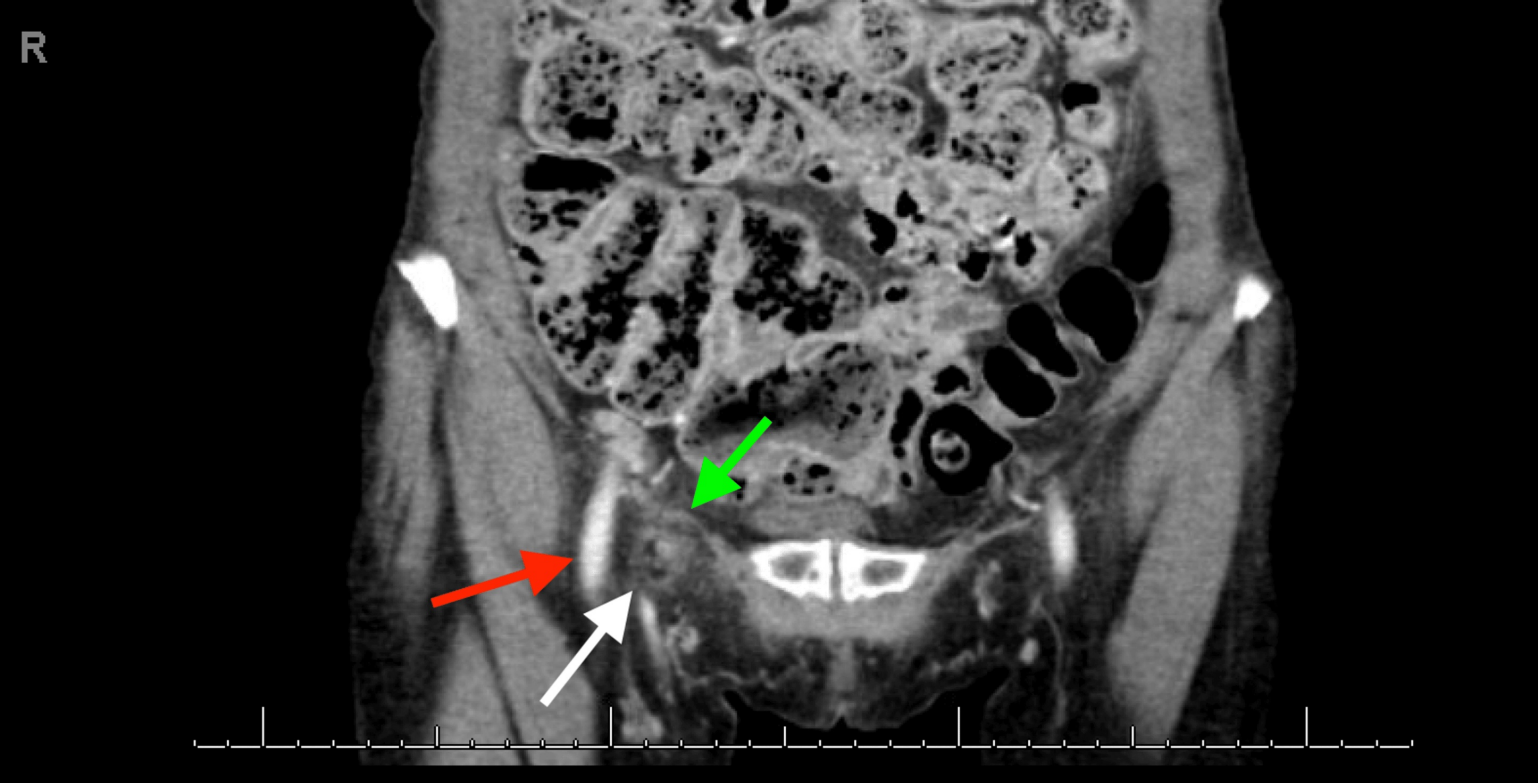
Contrast-enhanced computed tomography of the abdomen and pelvis Coronal view illustrating the appendix within the hernia sac (white solid arrow) in the femoral canal, medial to the femoral blood vessels (red solid arrow) and inferior to the inguinal ligament (green solid arrow).

A 63-year-old woman with a medical history of post-ablative hypothyroidism and bilateral salpingo-oophorectomy developed right groin pain and swelling following her routine daily exercise without any history of gastrointestinal symptoms or fever. The following day, the patient contacted her physician, who ordered imaging studies. US of the right groin demonstrates a hypoechoic lesion measuring approximately 2.04×1.78 cm with surrounding fluid and echogenic material, likely representing fat, anterior to the femoral blood vessels, as shown on the sagittal view in Figure 1, findings that were confirmed on subsequent contrast-enhanced CT. Figure 2 demonstrates the relative position of the hernia, which is anterior and medial to the right femoral artery and vein, seen on transverse view with color Doppler. Figure 3 shows the sonographic appearance of the normal contralateral groin for comparison. A contrast-enhanced CT of the abdomen and pelvis was urgently recommended for further assessment. CT demonstrates a right groin hernia at the level of the femoral vessels, with a hernia sac containing a dilated, fluid-filled tubular structure consistent with the appendix, which measures approximately 12 mm. There is wall thickening of the herniated portions of the appendix with adjacent fat stranding and edema. This was interpreted as a small right canal of Nuck hernia containing the tip of the appendix, findings consistent with acute appendicitis secondary to proximal obstruction. There is no perforation or abscess formation as demonstrated on the axial view in Figure 4, the sagittal view in Figure 5, and the coronal view in Figure 6. A summary of DGH imaging appearance is illustrated in Table 1.

**Table 1:** Summary of de Garengeot hernia imaging appearance

de Garengeot hernia imaging appearance	
Ultrasound	Hypoechoic, fluid-/air-filled tubular structure, +/- adjacent echogenic fat, within a hernia sac medial to the femoral vessels
Computed tomography	Confirms diagnosis with blind-ending tubular structure contiguous with the appendix, herniating medial to the femoral vessels and inferior to the inguinal ligament, inferior and lateral to the pubic tubercle, +/- compression of the adjacent femoral vein

Later that day, the patient visited the emergency department for further assessment. Physical examination revealed a tender, irreducible right inguinal mass, while laboratory tests were unremarkable. Subsequently, an urgent robot-assisted surgery was performed. Intraoperatively, it was found that the patient had a strangulated femoral hernia containing an inflamed distal appendix, compatible with a DGH complicated by acute appendicitis. An appendectomy and hernia repair were performed with preperitoneal mesh placement without any complications.

## Discussion

DGH containing an inflamed appendix is an unusual condition, accounting for 0.08-0.13% of all femoral hernias [3]. Most cases are diagnosed intraoperatively due to nonspecific symptoms, overlapping clinical and imaging features with different kinds of groin hernias, the rarity of these cases, and atypical presentation. The canal of Nuck is an abnormal continuation of the parietal peritoneum into the labia majora through the inguinal canal. Incomplete obliteration of this structure is termed patent processus vaginalis and can result in inguinal hernias in women [5]. There is limited awareness about this rarely occurring clinical entity in the medical community, and the exact incidence, varying clinical presentations, and complications associated with the canal of Nuck hernia remain unclear [6]. Ultimately, the index patient's case was determined not to be a canal of Nuck hernia, as the neck of the hernia originated inferior to the inguinal ligament, with the herniated contents contained within the femoral canal consistent with a DGH. Accurate radiological diagnosis is important and can help direct surgical exploration in the appropriate anatomic region, hence lowering the likelihood of complications, such as appendiceal ischemia, abscess formation, perforation, necrosis, necrotizing fasciitis, and small bowel obstruction [7]. Accurate sonographic diagnosis of hernia on US is often challenging due to technical limitations, variability in sonographer skill, and difficulty in delineating anatomic landmarks needed to correctly localize the hernia. However, it is still important to be familiar with the US appearance of an appendix-containing groin hernia to suggest such a diagnosis and ensure a time-sensitive follow-up CT scan to confirm the diagnosis, thus decreasing the risk of complications of acute appendicitis. According to the American College of Radiology, CT offers an overall sensitivity of 95% and an overall specificity of 94% for the diagnosis of appendicitis [8]. Multidetector CT scan is the best imaging modality for the evaluation and differentiation of groin hernias because it can easily demonstrate anatomic landmarks in the groin. Femoral hernias can be identified as they enter the femoral canal medial to the femoral blood vessels and inferior to the inguinal ligament. They commonly have a funnel-shaped neck appearance while occasionally compressing the femoral vein. Additional criteria that can aid with the differentiation of the femoral hernia from the inguinal hernia are based on the relative location of the hernia sac to the pubic tubercle, with the femoral hernia commonly appearing below and lateral to the pubic tubercle, while the inguinal hernia usually emerges above and medially [9].

## Conclusions

This case highlights the challenges associated with the radiological diagnosis of atypical presentations of groin hernias, especially in cases of DGH, in which inaccurate diagnoses through radiological imaging are prevalent. Knowledge of such cases and their typical imaging features not only allows for accurate diagnosis, thereby helping to guide surgical approach, but also allows for diagnosis to be made in a time-sensitive manner, possibly helping to reduce the risk of complications, such as in the setting of acute appendicitis.
